# A randomized controlled trial of cystoinflation to prevent bladder injury in the adhesive disease of multiple caesarean sections

**DOI:** 10.1038/s41598-020-71132-5

**Published:** 2020-09-17

**Authors:** Shazia Saaqib, Ayesha Iqbal, Munazza Naheed, Tayyaba Saeed, Mohammad Khalid

**Affiliations:** 1 King Edward Medical University/Lady Willingdon Hospital, Lahore, Pakistan; 2 Institute of Public Health, Lahore, Pakistan

**Keywords:** Anatomy, Health care, Urology

## Abstract

Caesarean sections carry the risk of urinary bladder injury due to formation of adhesions obscuring pelvic planes. Visualizing bladder during retro-fill (cystoinflation) makes it recognizable as it rises into the abdomen taking a tense rounded contour. We conducted a prospective randomized controlled trial to find out whether improved identification of bladder margins by cystoinflation could decrease bladder injury rate and blood loss without causing urological complications. This study included 214 healthy women with previous operative deliveries undergoing elective caesarean section and found to have dense pelvic adhesions. The subjects were randomly allocated into cystoinflation and control groups. Adhesiolysis was performed using bladder retro-fill with 300 cc saline in cystoinflation group, and without retro-fill in control. The bladder injury rate was significantly lower in cystoinflation group compared to control (2.8% vs 20.6%, P < .0001) with lesser blood loss in cystoinflation group (585.33 cc vs 797.10 cc, P < .0001). Mean operative time was similar in both groups. Urinary tract infection and micturition problems occurred more frequently in control group than cystoinflation group (16.8% vs 1.9%, P = .001 and .47 ± 1.63% vs 077 ± .633%, P = .021 respectively) with fistula in one subject compared to none in cystoinflation group. In this study, cystoinflation was effective to significantly reduce bladder injury rate and blood loss. This technique may also prove useful in the fields of surgery, urology and urogynecology.

## Introduction

The urinary bladder injury is a rare but important complication of caesarean section (C-section). The decreasing trend of vaginal birth after C-section has led to an increased number of women with multiple C-sections^1^. After caesarean delivery, adhesions may develop between bladder and uterus (24.4–73%) which cause difficulty in identification and dissection of the bladder flap during the next operative delivery^2,3^. Most of the bladder injuries occur at the time of opening the peritoneal cavity and during the creation of the bladder flap^4^. Bladder injury has been reported to occur with an overall incidence of 0.46% in a local Pakistani study, 0.44% in Saudi Arabia, 0.67% in Mumbai and 0.08–0.94% in international studies in C-section cases^5–10^. Tulandi and co-workers have proposed a scoring system of adhesions between bladder and uterus^3^.

**Table:** 

	Consistency of the adhesions	< 3 cm	3–6 cm	> 6 cm
Adhesions of previous C-section between uterus and bladder	Filmy	1	2	4
	Dense	4	8	16

Tulandi, T. & Lyell, D.J. Gynecol Surg (2013) 10: 25. https://doi.org/10.1007/s10397-012-0765.

Bladder injury has serious physical, social and psychological implications^11^. The injury leads to prolonged operative time, urinary tract infection (UTI), micturition problems and prolonged catheterization^3^. A long complicated hospital stay creates an intense feeling of helplessness and anxiety^11^. Moreover, unrecognized bladder perforation is a leading cause of morbidity due to the development of urinary ascites, peritonitis, and fistula formation^12–14^.

The adhesions are a major risk factor for bladder injury^15^. The routine practice of preoperative insertion of urinary catheter improves visualization in the operative field but fails to prevent bladder injury in dense adhesions^16^. Dye test with methylene blue is one of the methods used to identify bladder injury^3^. We observed that in women with intact bladder, it made the bladder outline prominent thus improving identification of bladder outline. This observation raised the question of whether improved identification of bladder margins by bladder inflation could decrease the occurrence of bladder injury in adhesions of previous C-sections.

In this study, we used cystoinflation (inflating bladder with saline retro-fill) to recognize bladder outline. In literature, there is insufficient evidence to support the use of cystoinflation to prevent bladder injury. The purpose of this study was to find the effectiveness and safety of cystoinflation to prevent bladder injury in women with adhesions of previous C-sections.

## Materials and method

This trial was approved by the ethical committee of King Edward Medical University (KEMU), Pakistan with IRB #216`/RC/KEMU on 27th March 2017 and registered at Clinical Trials.gov with the Reg#NCT04302545 on 10th March 2020, a primary register that participates in the WHO International Clinical trials registry platform. This study was designed according to the Declaration of Helsinki and followed the Good Clinical Practice Guidelines. We held this study at Lady Willingdon Hospital, a tertiary care teaching hospital affiliated with KEMU, Pakistan. The recruitment of this prospective study was carried out from 1st August 2017 to 30th April 2019 and follow up of each subject was conducted for a period of three months (follow up of last subject was completed on 31st July 2019).

### Sample size

Based on previous hospital statistics of our target population (women with adhesions), about 400 subjects were expected to present during the study period. The sample size was calculated to be 107 for each group at 95% confidence level and 80% power, allowing a 10% drop out rate (Raosoft, Inc 2004; online sample size calculator for proportions). The cystoinflation was considered to be effective if the proportion of subjects with bladder injury in the study group was less than 50% of the control.

### Randomization

The calculated sample size was randomized into the cystoinflation and control groups by the parallel assignment. Randomization was done by Principal surgeon by generating random numbers with the help of STAT TREK random number generator (downloaded from the internet). The study team members wrote the random numbers on closed envelopes with their corresponding groups inside and serially placed them in a file with the smallest number uppermost. The file was kept in the safe custody of theatre in-charge nurse who disclosed the group to the Principal surgeon after enrolment of subject in the study and provided setup for cystoinflation. The random number of each subject was entered on her documents including hospital admission file, consent form, trial entry form and outcome form by the study team medical officer in the theatre.

### Study criteria and selection of subjects

The subjects of the study were selected in two steps. In the first step, the women who met the preoperative study criteria and gave the preoperative informed consent were invited to participate in the study. In the second step, during operation, the women who fulfilled the inclusion criteria of adhesions were enrolled in the study.

Preoperative inclusion criteria were healthy pregnant women of any age, gestational age between 38 and 40 weeks (confirmed by dating scan) and two or more previous C-sections. Patients with the history of medical disorders, placenta previa and micturition problems (dysuria, frequency, urgency, urinary retention, incontinence) were excluded from the study.

The women admitted for elective C-section had detailed history, examination, preoperative investigations i.e. blood group, complete blood examination, urine examination and ultrasound scan. The women fulfilling preoperative study criteria were thoroughly counselled about cystoinflation. All the volunteers provided preoperative written informed consent to participate in the study if dense pelvic adhesions were found during their C-section and be assigned to either group of study. The subjects were selected in second step during C-section. The primary surgeons of the C-section were 2nd and 3rd-year residents of Obstetrics and Gynaecology. If dense adhesions were encountered, they discontinued and called the Principal surgeon (Assistant Professor Obstetrics and Gynaecology). The Principal surgeon categorized the adhesions according to Tulandi’s classification. The same surgeon (Principal surgeon) enrolled the subjects in the study if inclusion criteria of adhesions were fulfilled and assigned the subjects into the groups according to the random number envelope, performed adhesiolysis and completed the operation to avoid bias.

The perioperative criteria of adhesions for inclusion in the study were

- Dense pelvic adhesions of Tulandi score 4 and above.
- Adhesions were covering the bladder and lower segment of the uterus so that empty bladder could not be identified among the adhesions.
- Exteriorization of the uterus was not possible without adhesiolysis.
- Adhesions were encountered either at entry into the peritoneal cavity or before the creation of the bladder flap.

The women with bladder injury prior to enrolment were excluded from the study.

The group assignment was concealed from subjects, the medical and paramedical staff outside the operation theatre, outcome assessors, surgeons (before operation) and statistician of the study.

### The procedure of cystoinflation

#### Catheterization

All the women were catheterized before the operation under spinal anaesthesia. The theatre staff nurses were trained to perform catheterization with full aseptic measures. After scrubbing the abdomen and perineum of the woman with Povidone-iodine, the staff nurse cleansed her urethral meatus and perineum with sterile water soaked gauze pieces held with sponge holder- from front backwards- in the supine position with legs spread and feet together (using separate gauze piece each time). Then, one of the primary surgeons held the catheter tubing high above the perineum as the staff nurse inserted the catheter into urethra well beyond the catheter bulb without touching it elsewhere and held it there, while the surgeon inflated the bulb with 10 cc distilled water. The tubing was kept elevated whilst the theatre attendants straightened her legs and surgeon draped her with sterilized sheets. After that, surgeon fixed the urinary port end of the catheter to the drape in front of the thigh and attached it to the urine bag for drainage.

#### Cystoinflation and adhesiolysis

After random group assignment, the Principal surgeon performed adhesiolysis with an inflated bladder in cystoinflation group, and with an empty bladder in the control group. The cystoinflation was performed under strict aseptic measures. In the cystoinflation group, the assigned house officer retro-filled the bladder with 300 cc normal saline- with five fills of 60 cc bladder wash syringe- at a rate of 60 cc in one minute whilst the Principal surgeon identified the outline of gradually distending bladder among adhesions by looking into the pelvis. The urine port was then clamped with an artery clip and the surgeon performed adhesiolysis by sharp dissection keeping away from the bladder margins. After completing adhesiolysis, the house officer removed the artery clip and the bladder was emptied in a urine bag.

The subjects assigned to the control group had their adhesiolysis with the standard sharp dissection method routinely used in our hospital with urinary catheter put on free drainage. If there was a suspicion of bladder injury in either group, it was confirmed by performing the retrograde dye test with methylene blue and injury was repaired without delay by the Principal surgeon.

### Outcomes

Primary outcomes were the bladder injury rate, the extent of injury, operative time, blood loss and proportion of subjects receiving a blood transfusion. The bladder injury was detected by direct observation during surgery. The extent of the injury was analyzed by the size, site and depth of injury. The suspected bladder injury was confirmed with the dye test. The staff nurse recorded the operative time as time in minutes from incision to closure of the skin. The estimated blood loss was the increase in weight of sponges used during the operation, taking 1 g equal to 1 cc of blood.

Postoperatively, subjects were assessed for secondary outcomes of UTI, micturition problems, and fistula formation during the hospital stay and for the next 3 months. UTI markers were more than ten pus cells/hpf in urine and positive urine culture for infectious microorganisms on the third postoperative day. Dysuria was expressed by the subject as painful micturition using an eleven point Numeric Rating Scale ICCs (0.673–0.825), r = 0.7–0.99. (0 = No Pain, 1–3 = Mild Pain, 4–6 = Moderate Pain, 7–10 = Severe Pain). Postoperative micturition problems were feeling of incomplete evacuation, frequency, urgency, urethral and extra-urethral incontinence. The feeling of incomplete evacuation was defined as a feeling of fullness of the bladder with scanty micturition volume every time the patient tried to void. Urinary frequency was defined as more frequent desire to void, (8 times or more in 24 h period). The urgency was defined as a sudden compelling desire to void. Postoperative micturition problems were measured subjectively on a Likert scale questionnaire (0-never, 1-rarely, 2-sometimes, and 3-often) and analyzed as a composite variable during the hospital stay and for the next three months. Urinary retention was diagnosed if post-micturition bladder volume was greater than 50 cc on ultrasonography. The catheter was removed after twenty four hours in distension arms of both groups and subjects stayed in the hospital for four days. In the bladder injury arms, the catheter was kept in situ for seven days if only muscularis disruptions were repaired, for ten days if perforations smaller than1cm were repaired, and fourteen days if size of repaired perforation was greater than 1 cm. The subjects were discharged after twelve hours of catheter removal if there were no urinary complaints.

The subjects had to visit the study team for any micturition problem after discharge and their findings were entered on the follow-up form. For urinary incontinence, the fistula formation was ruled out by three swab test and retrograde cystography. The postoperative visit at the end of three months was mandatory in which all subjects were analyzed for postoperative micturition problems and their retrograde cystography was performed to rule out fistula. The subjects with fistula were referred to the urologist for further management. The outcome of each subject was noted on a Performa by assigned medical officers and entered in SPSS20 datasheet.

### Statistics

Statistical analysis was performed by using IBM spss statistics 20 (SPSS Inc, Chicago, IL, USA). The normality of continuous data was analyzed by the Shapiro Wilk test. The normal data of both groups was compared by T-test, non-normal data with Mann Whitney U test and categorical data with the chi-square test. The bladder injury of the groups was noted as numbers and percentages calculated by descriptive statistics. Both the groups were further divided into bladder injury arm and distension arms to analyze other primary outcome parameters. The effect of cystoinflation on outcome data was analyzed using regression analysis. The power of the study for bladder injury was 0.988, calculated with statistical software G’Power version 3.1.

## Results

**Figure 1:**
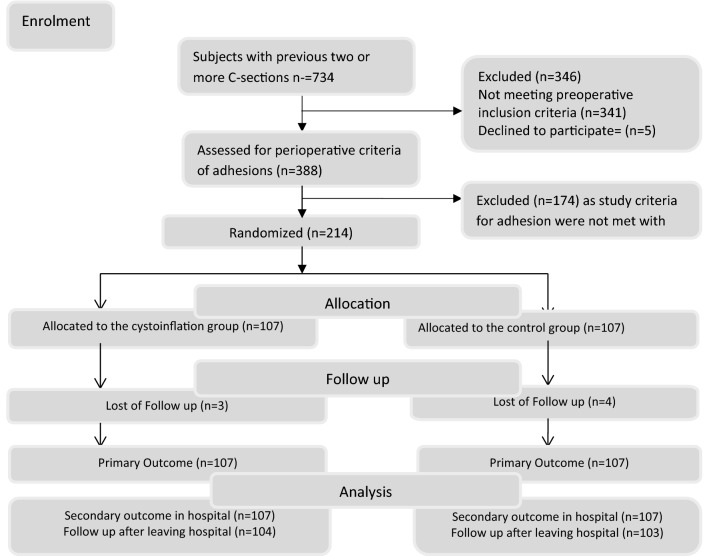
Consort diagram. Patient assignment to groups, follow up and analysis.

We conducted and reported this trial according to Consort Guidelines. From 1st August 2017 to 30th April 2019, we recruited two hundred and fourteen healthy pregnant women fulfilling inclusion criteria during the study period and conducted follow up of each subject for three months. The subjects were randomly allocated into cystoinflation group and control group by parallel assignment. Three participants in the cystoinflation group and four in the control group did not come for follow up after discharge from the hospital. We included them in the analysis of characteristics and outcome of the subjects during the hospital stay but excluded them from the analysis of postoperative outcome after discharge. The Enrolment of subjects in the study is shown in Fig. 1.

The mean age of subjects was 32.19 ± 2.3 years (range 26–38 years) in cystoinflation group and 31.73 ± 2.4 years (range 27–38 years) in the control group. The gestational age of women was between 37 and 40 weeks in both groups. Parity of subjects was ranging from two to five whereas the majority of women in both groups (60%) had previous three C-sections. Preoperative post-micturition bladder volume measurements were within normal ranges (10–30 cc) in both groups. Dense adhesions score (Tulandi score) included in the study were 4, 8 and 16 in both groups. The statistical analysis showed that there was no significant difference in the degree of adhesions between the groups (Table 1).

**Table 1:** Demographic features of the study population.

Demographic features	Cystoinflation group (n = 107)	Control group (n = 107)	P-value
Age(years)	32.19 ± 2.3	31.73 ± 2.4	.161
**Gestational age (weeks)**			
37	21 (19.6%)	19 (17.8)	.808
38	73 (68.2%)	72 (67.3%)	
39	10 (9.3%)	14 (13.1%)	
40	3 (2.8%)	2 (1.9%)	
**Parity**			
2	35 (32.7%)	33 (30.8%)	.867
3	60 (56.1%)	60 (56.1%)	
4	10 (9.3%)	10 (9.3%)	
5	2 (1.9%)	4 (3.7%)	
**BMI kg/m**^**2**^	30.73 ± 1.89	30.54 ± 1.86	.472
**Socioeconomic status**			
Low	39 (36.4%)	41 (38.3%)	.952
Medium	55 (51.4%)	54 (50.5%)	
High	13 (12.1%)	12 (11.2%)	
**Number of previous C-section**			
2	46 (43%)	43 (40.2%)	.975
3	52 (48.6%)	54 (50.5%)	
4	6 (5.6%)	7 (6.5%)	
5	3 (2.8%)	3 (2.8%)	
**Score of adhesions**			
4	53 (49.5%)	56 (52.3%)	.919
8	38 (35.5%)	36 (33.6%)	
16	16 (15%)	15 (14%)	
Preoperative postmicturition bladder volume (cc)	15 (10–30)	20 (10–30)	.234

Values are means ± SD, Median (range) and number (proportions). P-value of T-test, Mann Whitney U test and Chi-square test. Significant P-value < .05.

The bladder injury rate was significantly lower in cystoinflation group compared to control (2.8% vs 20.6%; P < 0.0001) and the size of injury was significantly smaller (.033 ± 0.22 vs 794 ± 1.66; P < 0.0001). The number of perforations was also lesser in cystoinflation group compared to the control group (2; 1.9% vs 12; 11.2%; P < 0.0001). The location of injury was higher in cystoinflation group favouring the easier and safer approach to repair (all 3 injuries were at the dome of the bladder in cystoinflation group versus the control group in which 18 injuries were at the dome of the bladder and 4 injuries were at the posterior bladder wall). The ureteric and bladder trigone injuries did not occur in this study.

The outcomes of three other primary outcome parameters i.e. operative time, blood loss and blood transfusions, were compared first between the two main groups, and then between their bladder injury arms and distension arms (no bladder injury arm). The operative time difference was insignificant if both groups were compared on the whole (45 ± 8.66 min in cystoinflation group versus 45 ± 16.99 min in the control group; p = 1), but significantly longer in cystoinflation group, if only distension arms of both groups were considered (44.66 ± 8.54 min in cystoinflation group vs 37.59 ± 6.44 min in control; P < 0.0001). Contrarily, the operative time was significantly longer in the bladder injury arm of the control group compared to the bladder injury arm of the cystoinflation group (73.64 ± 14.49 min vs 56.67 ± 2.89 min; P = 0.059).

The blood loss was significantly lesser in the cystoinflation group than the control group when comparing both the groups on the whole (585.33 ± 152.52 cc vs 797.10 ± 385.09 cc; P < 0.0001) and also when comparing their bladder injury arms (946.67 ± 378.46 vs 14.68.18 ± 221.95; P = 0.002) and distension arms (574.90 ± 131.33 vs 623.41 ± 162.29; P = 0.024). A lesser number of subjects required blood transfusions in cystoinflation group than control (4, 3.7% vs 24, 22.4%; P < 0.0001). (Table 2).

**Table 2:** Outcome of cystoinflation and control groups.

(a) Primary outcome				
Outcome parameters	Cystoinflation group(n = 107)	Control group (n = 107)	β (95%Confidence interval)	P-value
Bladder injury rate	3 (2.8%)	22 (20.6%)	− 0.276 (− 0.261–0.094)	< 0.0001
Size (cm)	0.033 ± 0.22	0.794 ± 1.66	− 0.307 (− 1.082–0.442)	< 0.0001
**Site**				
Dome of bladder	3 (2.8%)	18 (16.8%)	− 0.215 (− 0.317–0.113)	< 0.0001
Posterior bladder wall	0	4 (3.7%)		
**Depth**				
Muscularis partial	1 (0.9%)	8 (7.5%)	− 0.247 (− 0.587–0.180)	< 0.0001
Muscularis total	0	2 (1.9%)		
Perforation	2 (1.9%)	12 (11.2%)		
**Operative time (min)**				
Total subjects	45 ± 8.66	45 ± 16.99	< 0.0001 (− 3.634–3.634)	1
Distension arm	44.66 ± 8.541	37.59 ± 6.437	0.419 (4.864–0.9.287)	< 0.0001
Injury arm	56.67 ± 2.887	73.64 ± 14.49	− 0.383 (− 34.63–0.691)	0.059
**Blood loss during surgery (cc)**				
Total subjects	585.327 ± 152.52	797.10 ± 385.09	− 0.341 (− 290.706–132.845)	< 0.0001
Distension arm	574.904 ± 131.326	623.412 ± 162.29	− 0.164 (− 90.64–6.38)	0.024
Bladder injury arm	946.667 ± 378.46	1,468.18 ± 221.95	− 0.593 (− 826.637–216.393)	0.002
**Blood transfusions**				
Total subjects	4 (3.7%)	24 (22.4%)	− 0.277 (− 0.275–0.099)	< 0.0001
Distension arm	2 (1.9%)	2 (2.4%)	− 0.015 (− 0.046–0.037)	0.839
Bladder injury arm	2 (66.7%)	22 (100%)	− 0.533 (− 0.550– −0. 117)	0.004

(b) Secondary outcome				
WbC count/hpf	6.26 ± 1.003	7.36 ± 3.063	− .234 (− 1.708–0.479)	0.001
Urine c /s	2 (1.9%)	18 (16.8%)	− 0.257 (− 0.226–073)	< 0.0001
Fever	2 (1.9%)	13 (12.1%)	− 0.201 (− 0.171–0.035)	0.003
Composite micturition problems during the hospital stay	0.014 ± 0.108	0.131 ± 0.436	− 0.182 (− 0.202–0.031)	0.008
postmicturition bladder volume(cc)	15.56 ± 4.968	14.91 ± 4.952	0.066 (− 0.682–1.991)	0.336
Vasicovaginal fistula	None	1 (1%)	− 0.069 (0.028–0.009)	0.318
Duration of urinary catheterization	1.25 ± 1.626	3.14 ± 4.575	− 0.266 (− 2.813–0.963)	< 0.0001
Duration of hospital stay (days)	4.18 ± 1.156	5.50 ± 3.506	− 0.246 (− 2.021–0.614)	< 0.0001
Composite micturition problems after discharge	0.0048 ± 0.0345	0.0728 ± 0.226	− 0.207 (− 0.112–0.024)	0.003

Values are mean ± SD, and number (proportions). UTI: urinary tract infection, WBC/hpf: white cell count per high power field, c/s: culture and sensitivity, β: Co-efficiant of regression, P-value: regression analysis.

Postoperatively, urinary tract infections (UTI) and micturition problems occurred only in the bladder injury arm of both groups. Mean WBC count was higher in the control group than cystoinflation group (7.36 ± 3.063 vs 6.26 ± 1.003; P = 0.001) with more culture-positive cases of UTI (18, 16.8% vs 2, 1.9%; P < 0.0001) and fever (13; 12.1% vs 2; 1.9%; P = 0.003 respectively). The post-micturition bladder volume had no statistical intergroup differences (P = 0.336). In cystoinflation group, mean duration of urinary catheterization (1.25 ± 1.626 days vs 3.14 ± 4.575 days; P < 0.0001) and hospital stay (4.18 ± 1.156 days vs 5.50 ± 3.506 days; P < 0.0001) were also significantly shorter compared to the control group.

## Discussion

In this study, the bladder injury rate was seven times lesser in cystoinflation group compared to the control, thereby strongly supporting our hypothesis. The size of injury and amount of blood loss were also significantly decreased. Although the mean operative time was similar in the main groups, separate analysis of bladder injury arms and distension arms revealed significantly prolonged operative time in distension arm of cystoinflation group and bladder injury arm of the control group. Moreover, postoperative UTI and micturition problems were more common in the control group compared to cystoinflation group.

### Strength and limitations of the study

Cystoinflation or bladder retro-fill is a new technique to prevent bladder injury in Obstetric surgery. This study is among one of the earliest researches to observe the effect of cystoinflation in repeat C-section. The population of the study had confirmed dense adhesions increasing the validity of the study. Strength of study is high follow up rate, thereby decreasing bias in study results.

One of the study limitations was that it could be blinded only for the subjects. Blinding of the surgeon was unfeasible as identification of bladder wall is the cornerstone to prevent bladder injury. If the surgeon was unaware that the bladder is inflated, he could take wrong cleavage plane in the bladder wall instead of adhesions. Therefore, in a double-blind study, the rate of bladder injury could rather increase. Another study limitation was that the surgeon bias for detecting adhesions and performing adhesiolysis could not be ruled out as adhesiolysis in all cases was carried out by the same surgeon. Moreover, the question of optimum volume of the bladder filling could not be solved in this small study, as the effect of cystoinflation with different volumes of saline was not observed. Secondly, the best substance to use for cystoinflation (whether methylene blue, CO_2_ or normal saline) is still unknown. Cystoinflation requires consent, thorough counselling, and setup of retrograde filling along with the assignment of trained staff to perform the procedure. This technique is logistically difficult to implement during an emergency but may be helpful in elective cases. This study is a small single-centre trial that requires large, well designed, multicenter, controlled trials for generalization of results.

### Interpretation

Bladder injury is the commonest complication of multiple C-sections. With each subsequent C-section, the adhesions become denser and the risk of bladder injury is also increased (0.6% vs 0.19%)^16,17^. The attempts to prevent adhesion formation by different methods like hydroflotation, absorbable and non-absorbable membranes, derivative gel etc. are still under consideration and require further studies^18,19^. Surgeons are also contributing to minimize the complications of adhesions by modifying their surgical techniques. The omission of bladder flap formation has also been tried in this context but further evidence is required to prove its effectiveness^20,21^. The Bladder deflation by preoperative insertion of urinary catheter is a routine practice to prevent bladder injury during C-section^3,22^. However, recent studies are pointing out towards the safety of C-section without the catheter^16,23–25^.

In this study, bladder retrofill has proved useful to prevent bladder injury. A recent study of women with multiple C-sections, including cases of placenta previa, has also reported a significant reduction in bladder injury with a full bladder^16^. The same results have been observed in a retrospective study of laparoscopic gynaecological surgery with complete prevention of bladder injury with cystoinsufflation using CO_2_^26^_._ Nevertheless, this favourable effect of cystoinflation has not been observed in the caesarean hysterectomy of placenta accreta^17,27^. The poor visualization in the operative field due to increased vascularity and excessive bleeding in placenta previa cases may account for this difference, making cystoinflation less effective for creation of bladder flap.

Bladder injuries can still occur despite the successful creation of the bladder flap if the bladder adhesions are strongly attached to the anterior wall of the uterus^28^. If the pelvis is examined by the surgeon when the bladder is already full, presence or absence of bladder wall underneath the adhesion remains doubtful. However, if the surgeon is looking carefully into the pelvis during retrofill, the change in the shape of the adhesion during bladder filling will reflect the presence of a part of bladder beneath the adhesion and surgeon can avoid bladder injury even in the firmly adherent bladder by the correct demarcation of the bladder margins.

In our study, the bladder injury rate is very high in the control group (20%) compared to other studies of bladder injury in multiple C-Sections (0.6%)^10^; this difference can be partly explained by the difference in our study population. We included the most challenging cases of confirmed dense adhesions (Tulandi score 4 and above) which needed adhesiolysis to give incision in the lower uterine segment. According to the literature review, the risk of bladder injury is increased ten folds in multiple C-sections with adhesions^15,29^. However, the incidence of bladder injury exclusively with dense pelvic adhesions is not available. In the study of full bladder conducted on difficult C-section cases, the bladder injury rate was 18.4% in the control group which is quite comparable to our study^16^. Furthermore, the threshold of surgeon for dense adhesions, classification system used and expertise of the surgeon to perform adhesiolysis are other factors which may have influenced the incidence of bladder injury. This discrepancy can be solved by conducting further trials with same criteria of dense adhesions.

It has been observed that with cystoinflation, bladder injury is not decreased in cases of Placenta Previa but easy cutting and ligation of aberrant vessels decreases bleeding and shortens operative time^17,27^. Our study also shows a significant decrease in blood loss and blood transfusions with cystoinflation among the groups, their distension arms and the bladder injury arms. These results are also supported by the trial of full bladder in difficult C-sections^16^. The decrease of blood loss in both arms of the study may suggest that adhesiolysis in correct planes due to cystoinflation contributed to decreased vascular injuries.

In our study, the mean operative time is similar in both groups but significantly prolonged in distension arm of cystoinflation group compared to the control group distension arm. This finding is different from other studies of full bladder in which the operative time is shortened^16,17,27^. The difference may be due to the difference in readiness and speed to perform cystoinflation in different setups. In our study, even a small perforation (0.5 cm) of distended bladder led to a jet of fluid visible in the operative field restricting the size of injury to a minimum and injury was instantly recognized. This early detection shortened the time of repair in bladder injury cases, decreased blood loss and made the repair more successful and easier.

According to a literature review, 43% of bladder injuries occur at the creation of a bladder flap, 33% at entry into the peritoneal cavity, and 24% during uterine incision^3,28^. Based on this statement, bladder Injury is only likely to occur before delivery of the baby. In this study, we performed cystoinflation whenever dense adhesions were encountered, either before or after opening the peritoneal cavity, and it was always before delivery of the baby which may account for significant prevention of bladder injury in this study. Our results are supported by the study of full bladder before C-section^16^ whereas cystoinflation has failed to prevent bladder injury after deliveries of the baby in the study of placenta accreta^17^.

According to some studies, bladder distension can lead to urinary retention and other micturition problems but our results are quite contrary^30,31^. The possible explanation may be that the volume used for bladder retrofill in our study was 300 cc which lies within the limits of normal bladder capacity i.e. 300–400 cc^31^. It is discernible that this volume was adequate to make the bladder outline prominent but it did not distend the bladder wall to the extent to cause micturition problems.

Interestingly, UTI and micturition problems (the theoretical risks of cystoinflation) were observed only in the bladder injury arm of the control group, thereby supporting the safety of cystoinflation. Overall data from this study accounts for beneficial effects of cystoinflation to prevent bladder injury in repeat C-section. Novel strategies may help to shorten the time of bladder retro-fill, making it more useful in emergency cases.

Cystoinflation can also prove useful for separation of the bladder flap in cases of distorted pelvic anatomy due to large anterior wall fibroid, hysterectomies of previous pelvic surgeries and pelvic surgeries of other specialities like urology and general surgery. Nevertheless, further studies are required to determine whether such an approach is feasible and of real benefit.

## Data Availability

The datasets generated and analyzed during the current study are available from the corresponding author on reasonable request.

## Abbreviations

C-section: Caesarean section
OBG: Gynaecology and obstetrics
UTI: Urinary tract infection
ICC: Intra-class correlation coefficient for test retest reliability
r: Validity coefficient

## References

[1] Mumtaz S, Bahk J, Khang Y-H (2017). Rising trends and inequalities in cesarean section rates in Pakistan: evidence from Pakistan demographic and health surveys, 1990–2013. PLoS ONE.

[2] El-Mowafi, D., et al. Are pelvic adhesions preventable? europepmc.org. 12931305

[3] Tarney C (2014). Bladder injury during cesarean delivery. Curr. Womens. Health Rev..

[4] Rahman MS (2009). Bladder injuries during cesarean section in a university hospital: a 25-year review. Arch. Gynecol. Obstet..

[5] Kafali H (2008). Bladder injury during cesarean section. Arch. Gynecol. Obstet..

[6] Phipps MG, Watabe B, Clemons JL, Weitzen S, Myers DL (2005). Risk factors for bladder injury during cesarean delivery. Obstet. Gynecol..

[7] Rajasekar D, Hall M (1997). Urinary tract injuries during obstetric intervention. BJOG Int. J. Obstet. Gynaecol..

[8] Kaskarelis D (1975). Urinary tract injuries in gynecological and obstetrical procedures. Int. Surg..

[9] Morris S, Turocy J, Dabney L, Hardart A (2016). Bladder injury during cesarean delivery: a retrospective, 15-year study [28C]. Obstet. Gynecol..

[10] Eisenkop SM, Richman R, Platt LD, Paul RH (1982). Urinary tract injury during cesarean section. Obstet. Gynecol..

[11] Pinto A, Faiz O, Davis R, Almoudaris A, Vincent C (2016). Surgical complications and their impact on patients’ psychosocial well-being: a systematic review and meta-analysis. BMJ Open.

[12] Tai CK, Li SK, Hou SM, Fan CW (2008). Bladder injury mimicking acute renal failure after cesarean section: a diagnostic challenge and minimally invasive management. Surg. Laparosc. Endosc. Percutaneous Tech..

[13] Al-Mandeel H, Qassem A (2010). Urinary ascites secondary to delayed diagnosis of laparoscopic bladder injury. J. Minim. Access Surg..

[14] Ko PC, Lo TS, Ashok K (2011). Urinary ascites with elevated blood creatinine following cesarean section indicating bladder injury. Taiwan. J. Obstet. Gynecol..

[15] Korniluk A, Kosiński P, Wielgos M (2017). Intraoperative damage to the urinary bladder during cesarean section—literature review. Ginekol. Pol..

[16] Ali AE-N, Ahmed MAM, Khodry MM, Abbas AM (2019). Could bladder inflation prior to cesarean section prevent urinary tract injury in high risk group? A randomized controlled trial. Open J. Obstet. Gynecol..

[17] Özcan HÇ (2018). Use of bladder filling to prevent urinary system complications in the management of placenta percreta: a randomized prospective study. Geburtshilfe Frauenheilkd..

[18] Towfigh S, Clarke T, Yacoub W, Pooli AH, Mason RJ, Katkhouda N, Berne TV (2011). With daily probing of contaminated wounds. Arch. Surg..

[19] Molazem Z, Mohseni F, Younesi M, Keshavarzi S (2014). Aloe vera gel and cesarean wound healing; a randomized controlled clinical trial. Glob. J. Health Sci..

[20] O’Neill HA, Egan G, Walsh CA, Cotter AM, Walsh SR (2014). Omission of the bladder flap at caesarean section reduces delivery time without increased morbidity: a meta-analysis of randomised controlled trials. Eur. J. Obstet. Gynecol. Reprod. Biol..

[21] Youssef YA, Farghaly TA, Elsenosy E, Youssef AA, Abbas AM (2019). The effect of omission of the bladder flap formation at lower segment cesarean delivery: a randomized controlled trial. Open J. Obstet. Gynecol..

[22] Faricy P, Augspurger R (1978). Bladder Injuries Associated with Cesarean Section.

[23] Pandey D, Mehta S, Grover A, Goel N (2015). Indwelling catheterization in caesarean section: time to retire it!. J. Clin. Diagnostic Res..

[24] Abdel-Aleem H, Aboelnasr M, Habib F (2014). Indwelling bladder catheterisation as part of intraoperative and postoperative care for caesarean section (review). TheCochrane Libr..

[25] Li L, Wen J, Wang L, Li Y, Li Y (2011). Is routine indwelling catheterisation of the bladder for caesarean section necessary? A systematic review. BJOG An Int. J. Obstet. Gynaecol..

[26] O’Hanlan KA (2009). Cystosufflation to prevent bladder injury. J. Minim. Invasive Gynecol..

[27] Matsubara S (2018). Filling the bladder at cesarean hysterectomy for placenta percreta. Geburtshilfe Frauenheilkd..

[28] Franchi M (2019). Unintentional transvesical caesarean section: incidence, risk factors, surgical technique and post-operative management. Eur. J. Obstet. Gynecol. Reprod. Biol..

[29] Gungorduk K, Asicioglu O, Celikkol O, Sudolmus S, Ark C (2010). Iatrogenic bladder injuries during caesarean delivery: a case control study. J. Obstet. Gynaecol. (Lahore).

[30] Joelsson-Alm E, Nyman CR, Svensén C, Ulfvarson J (2014). Micturition problems after bladder distension during hospitalization in Sweden. Nurs. Res..

[31] Agrawal K, Majhi S, Garg R (2019). Post-operative urinary retention: review of literature. World J. Anesthesiol..

